# A community affair in the tumor microenvironment

**DOI:** 10.18632/oncotarget.22586

**Published:** 2017-11-21

**Authors:** Jeffrey J. Rodvold, Santosh Kesari, Maurizio Zanetti

**Affiliations:** Maurizio Zanetti: The Laboratory of Immunology, Department of Medicine and Moores Cancer Center, University of California at San Diego, La Jolla, CA, USA; Department of Translational Neurosciences and Neurotherapeutics John Wayne Cancer, Institute and Pacific Neuroscience, Institute Saint John’s Health Center, Santa Monica, CA, USA

**Keywords:** transmissible ER stress, UPR, tumor heterogeneity, chemoresistance, UPR intervention

Tumors are a mosaic of heterogeneous cell types, whose growth dynamics depend on various cell-intrinsic and cell-extrinsic events. The latter form the essence of what can be defined as the community effect, implying that the growth characteristics of the tumor are dictated by non-cognate intercellular interactions. The generation of this type of functional tumor heterogeneity was previously ascribed to cell non-autonomous effects, though the underlying cellular processes of the phenomena remained incompletely identified [1]. New findings from this laboratory propose a form of intercellular communication that relies on endoplasmic reticulum (ER) stress signaling [2]. In this paradigm, cancer cells undergoing ER stress release molecules that initiate de novo ER stress in otherwise nonstressed cancer cells, substantially modifying the population dynamics to confer resistance upon subsequent metabolic, pharmacologic, and genotoxic stresses. Collectively, ER stress-based intercellular communication creates a synergism that promotes clonal cooperation, much resembling bacterial *quorum sensing*, and survival advantage.

The fulcrum of this activity is the unfolded protein response (UPR), a phylogenetically conserved adaptive response to ER stress. The UPR is mediated by three initiator/sensor ER transmembrane molecules: inositol-requiring enzyme 1 (IRE1), PKR-like ER kinase (PERK), and activating transcription factor 6 (ATF6), which are repressed through association with the chaperone 78 kDa glucose-regulated protein (GRP78) during homeostatic proteostasis. When client proteins accumulate in the ER, GRP78 dissociates from these transmembrane proteins, activating downstream signaling to normalize protein folding and secretion through the activation of ER stress responsive genes. Notable concomitant effects also consist in the selective inhibition of translation to reduce ER client protein burden and facilitate a homeostatic balance of cellular functions. Thus, the UPR orchestrates the cellular response to exogenous (nutrient starvation, hypoxia, etc.) and endogenous (defective glycosylation, aneuploidy, etc.) stressors that may be native to the tumor microenvironment (TME) [3]. Not surprisingly, cancer cells rely on cell-intrinsic UPR signaling for survival advantage in the TME where unfavorable conditions abound. Surprisingly, we found that the UPR can also operate as a cell-nonautonomous phenomenon where ER stress is propagated from one cell to another cell.

Upon ER stress transmission, receiver cancer cells were found to develop tolerance to subsequent metabolic, pharmacological, and genotoxic stressors, with survival gains observed both in vitro and in vivo. Receiver cells also underwent several other adaptive changes including the transcriptional upregulation of pro-inflammatory cytokine genes, the activation of Wnt signaling, and the cytoplasmic enrichment of telomerase reverse transcriptase (TERT), features which have been independently implicated in driving chemoresistance and tumorigenicity [4, 5].

The UPR machinery appeared to be durably altered in receiver cells, with particular effects on the PERK pathway and GRP78. Upon ER stress transmission, receiver cancer cells decreased expression of both PERK and its downstream effector ATF4, during homeostatic as well as stress conditions. Since PERK activation leads to the transcription and translation of ATF4, which drives expression of the pro-apoptotic target CHOP [3], we concluded that ATF4 functions as a rheostat during transmissible ER stress adaptation, gauging the effects of transmissible ER stress in receiver cells to ultimately control cell fate (survival *vs.* apoptosis). Strikingly, receiver cancer cells also displayed increased GRP78 expression intracellularly and at the cell surface, which persisted for over one week. Given the wealth of reports linking GRP78 to tumor cell survival [6], cytoprotection by intercellular stress transmission may be the result of non-mutually exclusive mechanisms involving PERK and GRP78.

These studies demonstrate that UPR-based intercellular communication could drive functionally-heterogeneous tumor cell clones. Differential expression of UPR genes and proteins (GRP78, PERK, etc) may account for differential clonal survival in the same tumor. However, no clinical studies exist to confirm the conclusion that, independent of driver mutations, tumors cells have differential expression of the UPR. Conceptually, intercellular communication through UPR-based signaling represents a new mechanism to regulate population dynamics and contribute to intra-tumor heterogeneity, allowing neighboring cells to acquire growth advantage when selective pressures (e.g., nutrient starvation or chemotherapy) emerge.

What are the therapeutic implications of these findings? The dynamics and function of different cell types in the TME pose considerable challenges for cancer therapy, including chemotherapy and immunotherapy. Earlier experiments indicate that transmissible ER stress drives direct immunomodulation of innate immune cells, macrophages and dendritic cells, and indirect modulation of T cells during their activation from naïve to effector stage [7]. Therefore, intervening against the UPR may be a worthwhile target to undercut processes that endow chemoresistance as well as effecting innate immune cells. UPR-targeted interventions can be broadly stratified as either i) to inhibit downstream signaling, or ii) to generate unresolvable ER stress to drive apoptosis. The former category includes small molecules such as IRE1 and PERK inhibitors (reviewed [8]). The latter category includes drugs such as the protease inhibitor bortezomib, arsonate trioxide, and thapsigargin analogues (e.g., mipsigargin). What remains to be settled is how effective these drugs really are at dysregulating UPR signaling in preclinical cancer models and later in clinical trials.

Assessing the multiple effects, and associated mechanisms of action, on different cellular targets in the tumor microenvironment in response to intercellular communication of ER stress may be key to future therapeutic interventions. For this to be effective, attention should be paid to specific signaling targets in relation to tumor type and history, immune phenotype of infiltrating cells, genomic heterogeneity, and growth dynamics. Targeting intercellular ER stress transmission, and broadly the UPR, may become part of a future interventions in association with chemotherapy and immunotherapy.
